# Impact of COVID-19 pandemic on migraine management in the United States: insights from migraine tracking app users

**DOI:** 10.1186/s12883-021-02378-3

**Published:** 2021-09-09

**Authors:** Yuji Kato, Weijie Poh, Zsolt Horvath, François Cadiou, Tomokazu Shimazu, Yuichi Maruki

**Affiliations:** 1 Department of Neurology, Saitama Neuropsychiatric Institute, 6-11-1 Honmachi-Higashi, Chuo-ku, Saitama, Saitama 338-8577 Japan; 2 Department of Neurology and Cerebrovascular Medicine, Saitama Medical University International Medical Center, 1397-1 Yamane, Hidaka, Saitama 350-1298 Japan; 3 Healint Pte. Ltd, Singapore, Singapore

**Keywords:** COVID-19, Pandemic, Migraine, Real world, Smartphone application

## Abstract

**Background:**

The nature of COVID-19 pandemic measures has altered the clinical management of migraine, and has also created barriers to evaluate the impact of such measures of migraine patients. Using the Migraine Buddy smartphone application, we assessed the impact of the COVID-19 pandemic on migraine in users residing in the United States.

**Methods:**

Migraine Buddy is a smartphone application by individuals to record their migraine headache episodes, characteristics, and coping mechanisms. For this study, anonymized self-reported data from 163,176 adult Migraine Buddy users in the United States between January 2020 and May 2020, were analyzed for migraines associated with stress. A stress-related migraine is defined as one in which stress or anxiety was reported as a trigger or symptom. A questionnaire on the impact of COVID-19 on migraine and its management was also completed by 923 users from the United States in the app between April 2020 and May 2020.

**Results:**

88% of the Migraine Buddy database extract and 84% of the respondents are female, with a mean age of 36.2 years. The proportion of stress-related migraine attacks peaked at 53% on March 21 to 23, although the number of migraine attacks decreased. This followed the declaration of the COVID-19 national emergency on March 13 and a spike in the number of COVID-19 cases in the United States. Questionnaire respondents felt that the following added more stress: social isolation (22.6%), information overdose (21.2%), access to essentials (food, medication, etc.) (18.7%), and financial concerns (17.8%). To help manage migraine during COVID-19, respondents suggested stress and diet coaching programs and resources (medical articles, etc.) (34.0%), having the option for home delivery of medication (30.6%) and tele-consulting (25.5%).

**Conclusion:**

Here, we report the change in the proportion of self-reported stress-related migraine in relation to evolution of the COVID-19 pandemic, as well as its impact of migraine management. Our data will help increase the understanding of patients’ needs and help with planning and execution of mitigating strategies.

## Background

The World Health Organization declared COVID-19 a global pandemic on March 11, 2020. At the time of writing (July 2021), there have been 33,530,880 confirmed cases and 603,018 deaths in the United States [1]. New York City went into lockdown on March 22, 2020, and implemented stay-at-home measures for residents. As of May 30, 2020, New York has been the virus’ epicenter in the United States with 23,780 deaths and 368,234 cases [2]. Under such conditions, it is difficult to assess the impact of COVID-19 for migraine patients without face-to-face consultations.

With the recent developments in information technology, real-world big data studies have attracted increasing attention in the field of medicine. Gaining an insight into patient-reported outcomes is an important part of the wider scope of real-world medical data, and its standardized use is critical for the generation of high-quality real-world evidence to improve the core functioning and competitiveness of clinical care data as well as provide high-quality medical services for patients.

Migraine Buddy is the most widely used smartphone application by individuals worldwide to record their patterns of migraine headaches, characteristics (triggers, pain intensity, symptoms) and coping mechanisms (medication intake, relief methods) [3]. By tracking the migraine headaches over time, users and their headache specialists are able to better understand and manage the condition. Through the app, Migraine Buddy users also provide real-time insights via surveys and their diary records, which allows the Healint data analytics platform to extract key anonymized aggregated findings. Using the Migraine Buddy app, this study aimed to assess impact of the COVID-19 pandemic on migraine in Migraine Buddy users who reside in the United States.

## Methods

The study is a non-interventional, retrospective analysis of data that is self-reported via the Migraine Buddy smartphone application. Participants provided authorization for their data to be used for research, and deidentified data were used and analyzed for this study. Participants have the option to refuse to this condition and are able to use the app without any restriction if they choose to do so. As such, study subjects were not placed at risk by being included in the study sample, and the study was approved by the Institutional Ethics Committee at the Saitama Neuropsychiatric Institute (approval number: SNI 20–006).

A retrospective analysis was conducted using data captured through the smartphone application Migraine Buddy that is used for tracking migraine, tension headaches, and cluster headaches. For this study, only self-reported migraine records are included in the analysis. In the app, users are prompted to provide the following: attack duration, attack type, pain intensity, location of pain onset, medication taken, relief methods, symptoms, aura, prodrome, affected activities, location when the attack occurred, along with weather and sleep patterns collected by the app.

For this study, anonymized self-reported data from 163,176 adult Migraine Buddy users in the United States over the 5-month period from January 1, 2020 through May 31, 2020 were extracted from the Migraine Buddy database. Although a confirmed migraine diagnosis is not required from these patients, the large dataset provides a reasonable approximation to the responses from individuals living with migraine. The variables examined included the following: demographic characteristics (age, sex), migraine frequency and triggers (such as stress, lack of sleep, depressed mood, anxiety, skipped meals, smells, light, weather, neck pain, foods, caffeine, alcohol, dehydration etc). Each migraine record represents a single migraine attack. A stress-related migraine is defined as one in which stress or anxiety were self-reported as a trigger or symptom in the Migraine Buddy records. The cause of the stress trigger was not collected, and hence may or may not be due to COVID-19. The stress-related migraine group includes individuals who reported at least 1 stress-related migraine in each calendar month of the study period. The change of Dow Jones Industrial Average (DJIA) and the number of new COVID-19 cases in the United States were used as reference indexes [1].

In addition to the Migraine Buddy records, a questionnaire survey was sent to Migraine Buddy users in the United States, and completed in the app between April 2020 and May 2020. The survey was designed by authors to obtain responses of the impact of COVID-19 on migraine and migraine management during this period.

## Results

### Study population

The study sample included 1,116,605 migraine records from 163,176 users in the United States. 83% of users were female, with a mean age of 36.2 years. A summary of demographics and migraine characteristics of the study population is shown in Tables 1 and 2. The stress-related migraine group had a higher proportion of women than the migraine group that did not reported stress-related migraine (*p* < 0.0001). Although there were no difference in pain intensity and monthly headache days between the two groups, the usage rate of acute and preventive treatment was higher in stress-related migraine group than non- stress-related migraine group (*p* < 0.0001).

**Table 1:** Demographics and migraine information of users included in the study

	Overall (***N*** = 163,176)	Stress-related migraine (***N*** = 84,740)	Non stress-related migraine (***N*** = 78,436)	***p***
**Demographics**				
Age (years), mean (SD)	36.2 (12.6)	36.2 (12.4)	36.2 (12.9)	
Sex: number (%)				< 0.0001
Male	13,009 (12%)	6770 (10%)	6239 (14%)	
Female	95,512 (88%)	57,815 (90%)	37,697 (86%)	
Unknown	54,655	20,155	34,500	
**Migraine characteristics**				
Pain intensity, mean (SD)	5.4 (2.1)	5.4 (2.1)	5.5 (2.1)	
Monthly headache days, mean (SD)	5.3 (5.7)	5.7 (5.7)	4.6 (5.5)

**Table 2:** Migraine treatment of users included in the study

	Overall (***N*** = 163,176)	Stress-related migraine (***N*** = 84,740)	Non stress-related migraine (***N*** = 78,436)	***p***
**Medical characteristics**				
Acute treatment (OTC)				
Total, number (% of subjects)	102,676 (63%)	62,325 (74%)	40,351 (48%)	< 0.0001
Ibuprofen, number (% of reported)	57,032 (56%)	36,163 (58%)	20,869 (52%)	
Acetaminophen, number (% of reported)	56,704 (55%)	36,164 (58%)	20,540 (51%)	
Naproxen, number (% of reported)	15,656 (15%)	10,370 (17%)	5286 (13%)	
Acetylsalicylic acid, number (% of reported)	4074 (4%)	2690 (4%)	1384 (3%)	
Ketorolac, number (% of reported)	3684 (4%)	2538 (4%)	1146 (3%)	
Acute treatment (prescription)				
Total, number (% of subjects)	65,846 (40%)	42,056 (50%)	23,790 (28%)	< 0.0001
Sumatriptan, number (% of reported)	27,390 (42%)	17,684 (42%)	9706 (41%)	
Rizatriptan, number (% of reported)	17,347 (26%)	11,150 (27%)	6197 (26%)	
Ondansetron, number (% of reported)	5474 (8%)	3914 (9%)	1560 (7%)	
Eletriptan, number (% of reported)	4364 (7%)	2847 (7%)	1517 (6%)	
Zolmitriptan, number (% of reported)	4349 (7%)	2827 (7%)	1522 (6%)	
Preventive treatment				
Total, number (% of subjects)	27,029 (16.6%)	17,993 (21%)	9036 (11%)	< 0.0001
Topiramate, number (% of reported)	7950 (29%)	4980 (28%)	2970 (33%)	
Erenumab, number (% of reported)	3721 (14%)	2489 (14%)	1232 (14%)	
Botox, number (% of reported)	2532 (9%)	1803 (10%)	729 (8%)	
Galcanezumab, number (% of reported)	2250 (8%)	1594 (9%)	656 (7%)	
Propranolol, number (% of reported)	1899 (7%)	1310 (7%)	589 (7%)	

### Recorded migraine attacks

**Fig. 1:**
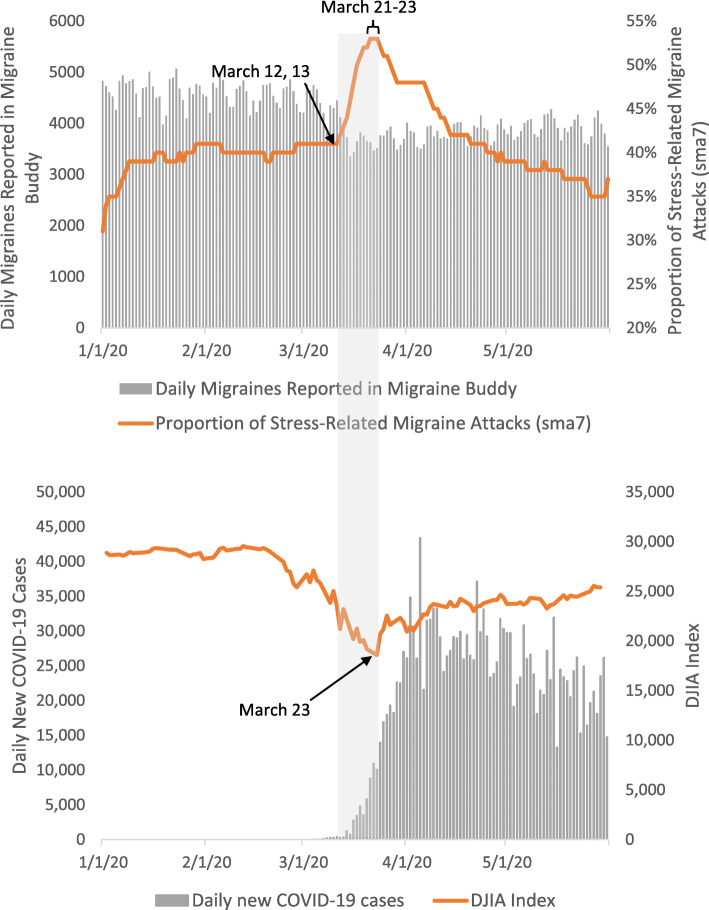
The graph shows trend about number of recorded headache attacks, the 7 day smooth moving aveage (sma7) ratio of stress-related headache attacks, Dow Jones Industrial Average and the number of new COVID-19 cases over time. The ratio of stress-related headache attacks reached the peak value of 53% on March 21 to 23, though the number of recorded headache attacks decreased. During this time, Dow Jones Industrial Average index decreased sharply

Figure 1 shows the number of recorded migraine attacks in the United States from January 1, 2020 to May 31, 2020. The number of recorded migraine attacks and proportion of stress-related migraine follow a consistent pattern when there is a sharp decrease on Saturday and Sunday, followed by an increase on Monday. On March 13, 2020, a “national emergency” was declared due to the COVID-19 pandemic followed by a spike in the number of new cases of COVID-19 in the United States. The proportion of stress-related migraine attacks recorded in Migraine Buddy reached the peak value of 53% on March 21 to 23, although the number of recorded migraine attacks decreased. During this time, DJIA index decreased by 37% from 29,551 points on February 12 to 18,591 points on March 23. The number of recorded migraine attacks gradually increased after April, but the number did not reach the figure recorded in March. On the other hand, the proportion of stress-related migraine gradually decreased.

### Questionnaire survey

**Fig. 2:**
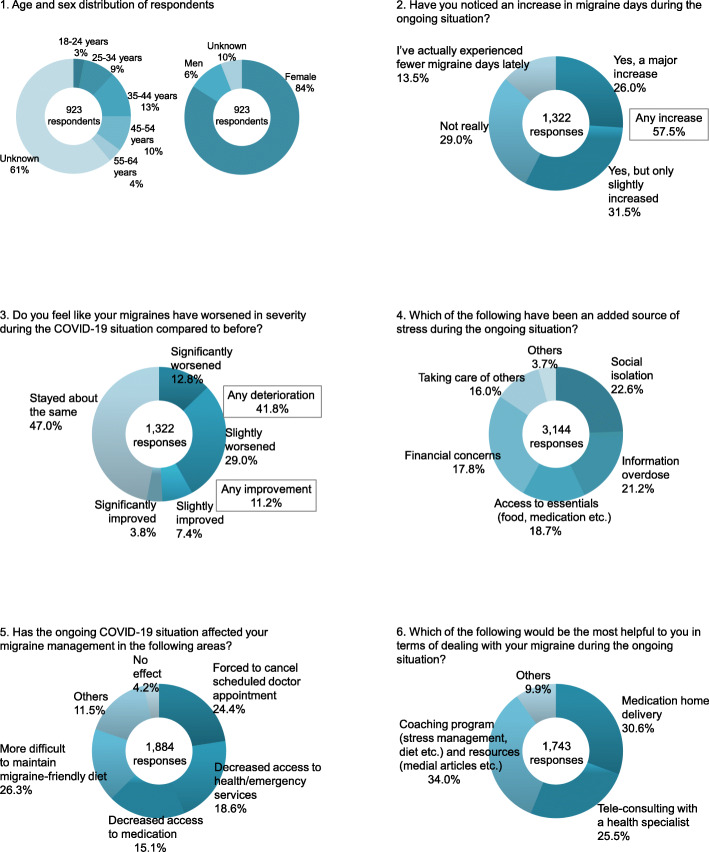
Results of questionnaire survey that was answered in April, May 2020 by 923 Migraine Buddy users in the United States

A total of 923 respondents in the United States completed the questionnaire survey, of which 84% is female; 13.0% were aged 35 to 44 years, 9% were aged 25 to 34 years, and 10% were aged 45 to 54 years (Fig. 2**-**1).

57.5% of respondents reported an increase in migraine days, with 26.0% describing a major increase (Fig. 2**-**2). Migraine severity was reported as worse compared to before the pandemic by 41.8% of respondents, with 12.8% describing this as significant (Fig. 2–3). Sources of stress were reported as follows: social isolation (22.6%), information overdose (21.2%), access to essentials (food, medication etc.) (18.7%) and financial concerns (17.8%) (Fig. 2–4). In terms of migraine management, more than half of responders (58.1%) experienced an interruption of medication, health/emergency services, and doctor consultations, with a quarter of responders (26.3%) experiencing difficulty in maintaining migraine-friendly diet (Fig. 2–5). According to the respondents, the most helpful way in managing migraine was having a coaching program (stress management, diet etc.) and resources (medical articles etc.) (34.0%), followed by medication home delivery (30.6%) and tele-consulting with a health specialist (25.5%) (Fig. 2–6).

## Discussion

In the present study, we reported an increase in the proportion of stress-related migraine at the beginning of the COVID-19 pandemic. The difference in the number of migraine attacks from to the Migraine Buddy records and those reported in the survey can be attributed to the fact that the survey was answered during April and May, whereas the biggest increase in stress-related migraine occurred in March. Similar to our findings, an online survey conducted in Kuwait demonstrated that the majority of respondents had reported increase in migraine frequency and severity during pandemic period [4].

Stress is known as the most common trigger of migraine attacks [5]. The COVID-19 pandemic, as a global health crisis, is perceived as a major stressful event. In a recent study from Spain, 41% of participants reported feeling moderate to severe stress and depressive symptoms during the pandemic, and 25% experienced mild to severe levels of anxiety [6]. Young women, who consisted of the majority of respondents in our study, felt the strongest negative impact [6]. COVID Stress Scales [7], which are scales measuring COVID-related stress and anxiety symptoms, may offer promise as tools for better understanding patients with COVID stress-related migraine.

Next, information overdose was reported as the second most common source of stress during pandemic in our questionnaire, with another study demonstrating that attention paid to COVID-19 media coverage as a risk factor for psychological distress in migraineurs [8]. This suggests that migraineurs could avoid paying too much attention on the media coverage of the pandemic.

Most respondents experienced significant life changes during a short period of time. In the light of questionnaire survey, respondents had difficulty maintaining migraine-friendly diets, scheduling doctor consultations, and accessing health/emergency services. A recent study reported that disturbance of eating habits was one of the factors affecting increase migraine frequency and severity [4]. A recent survey of 155 countries conducted by the World Health Organization found that nearly half of the patients with noncommunicable diseases failed to receive their regular medical care and medications since COVID-19 pandemic began [9]. With the various lockdown measures and shifts in healthcare systems towards COVID-19, it is plausible that this has resulted in a shift in patient behaviour towards self-management and may have exacerbated migraine.

Interestingly, from our questionnaire, 13.5% of respondents reported a reduction in migraine frequency and 11.2% reported improvement of migraine severity during pandemic period. This could be explained by the reduction in work-related pressure as workplaces shift towards a working from home arrangement, and backed up by our observation that migraine records decrease during the weekend. This is similar to recent studies from Italy and the Netherlands that reported fewer migraine attacks and lesser pain [10–12]. This study looking at the effect of COVID-19 lockdown on migraine provides interesting insights that working from home may have contributed to improvement in migraine for some individuals [11]. On the other hand, a recent study of Italian working women revealed that most of the additional housework and childcare duties associated with the COVID-19 pandemic were managed on predominantly by females, which coincidentally may be an added source of migraine trigger [13].

Interestingly, the ratio of stress-related migraine showed inverse correlation with the DJIA index. Rising fears and global economic shutdown due to the COVID-19 pandemic are believed to be main causes of the stock market crash [14]. During the study observation period, we observed that the bottom of the DJIA index overlaps with the peak of the ration stress-related migraine, suggestive that the stock market performance has an impact on stress-related migraine.

The ongoing threat of COVD-19, or other future pandemics, will require strategies for patients to better manage their migraine. The solutions identified by the survey respondents include coaching programs, medication home delivery and telemedicine consultation. Thus, it is a positive sign that organizations such as the American Migraine Foundation remain dedicated to providing support, education and comfort to those with migraine [15]. These resources will be benefits for patients managing migraine during these difficult times. Headache specialists should also consider taking steps to rapidly develop remote teleconsultation services. There is an urgent need to identify clinicians who are trained in telemedicine, expand existing remote consultation technologies with community hospitals and emergency departments and offer medication home delivery. Nonetheless, migraine management may still require in-person visits for infusions or procedures. It is reported that the cancellation of Botox injection sessions had a negative impact on migraine patients during the pandemic [4, 16]. In this regard, specialists can consider the use of other preventive therapies such as self-injectable monoclonal antibodies, neuromodulation devices and oral migraine prophylaxis medication [4, 16].

There are known limitations inherent to these real-world studies that rely on self-reported data from a smartphone app. First, there is potential bias as the study analyzed data reported by users, and no physician diagnosis is available to confirm a migraine diagnosis. However, the majority of respondents have reported the use of common migraine medication within the Migraine Buddy app.

Secondly, selection bias may arise owing to the requirement to have access to a mobile smartphone and comfort level in operating digital apps and, therefore, the population may not be entirely representative of the migraine population. Our respondents were unbalanced for sex and age, as the population to be young females. However, migraine is known to be more common in females, with the highest prevalence in the 20–50 age groups. Also, younger population usually constitutes the majority of smartphone app users.

Thirdly, being an observational study conducted via a smartphone app, the establishment of a direct causal relationship between migraine and COVID-19 pandemic can be difficult.

Lastly, we lack information of physical activity or dietary habits of the study subjects during the pandemic. These lifestyle elements may have an impact on patients during the pandemic [17]. Our goal is to present findings that can be representative of the real-world impact of COVID-19 on patients with migraine, given the rather large sample size. This will help increase the understanding of patients’ needs, and help with the planning and executive of mitigating strategies.

During COVID-19 pandemic, the clinical management of migraine as well as mental health should not be neglected. The stress-related psychosocial impact of the pandemic is evident and complex. Migraine attacks can be associated with a decline in perceived stress [18]. It is plausible to experience an increase in migraine cases once the COVID-19 pandemic situation has stabilized. Long-term follow-up data are necessary and smartphone apps like the Migraine Buddy can play a big role in monitoring changes in migraine managemnet. In future, the analysis of information about the body, collected via wearables, together with self-reported data entered into the migraine diary, can be performed by artificial intelligence to provide migraine patients with feedback to optimize their migraine management.

## Conclusion

Here, we leveraged the Migraine Buddy smartphone app to query and report the change in the proportion of self-reported stress-related migraine as the COVID-19 pandemic evolves, as well as its impact of migraine management. From these responses, we propose that headache specialists continue to develop appropriate strategies to help patients manage migraine and mental health. Our data will help increase the understanding of patients’ needs and help in the planning and execution of such mitigating strategies.

## Data Availability

The data generated for this study is available on reasonable request to the corresponding author.

## Abbreviations

DJIA: Dow Jones Industrial Average

## References

[1] Centers for Disease Control and Prevention. [online]. Available at: https://covid.cdc.gov/covid-data-tracker/#trends_dailytrendscases

[2] Dong E, Du H, Gardner L (2020). An interactive web-based dashboard to track COVID-19 in real time. Lancet Infect Dis.

[3] Vo P, Paris N, Bilitou A, Valena T, Fang J, Naujoks C, Cameron A, de Reydet de Vulpillieres F, Cadiou F (2018). Burden of migraine in Europe using self-reported digital diary data from the migraine buddy© application. Neurol Ther.

[4] Al-Hashel JY, Ismail II (2020). Impact of coronavirus disease 2019 (COVID-19) pandemic on patients with migraine: a web-based survey study. J Headache Pain.

[5] Sauro KM, Becker WJ (2009). The stress and migraine interaction. Headache.

[6] Rodríguez-Rey R, Garrido-Hernansaiz H, Collado S (2020). Psychological impact and associated factors during the initial stage of the coronavirus (COVID-19) pandemic among the general population in Spain. Front Psychol.

[7] Taylor S, Landry CA, Paluszek MM, Fergus TA, McKay D, Asmundson GJG (2020). Development and initial validation of the COVID stress scales. J Anxiety Disord.

[8] Ma M, Fang J, Li C, Bao J, Zhang Y, Chen N, Guo J, He L (2020). The status and high risk factors of severe psychological distress in migraine patients during nCOV-2019 outbreak in Southwest China: a cross-sectional study. J Headache Pain.

[9] World Health Organization (2020). Preliminary results: rapid assessment of service delivery for noncommunicable diseases during the COVID-19 pandemic.

[10] Parodi IC, Poeta MG, Assini A, Schirinzi E, Del Sette P (2020). Impact of quarantine due to COVID infection on migraine: a survey in Genova, Italy. Neurol Sci.

[11] Verhagen IE, van Casteren DS, de Vries LS, Terwindt GM (2021). Effect of lockdown during COVID-19 on migraine: a longitudinal cohort study. Cephalalgia..

[12] Delussi M, Gentile E, Coppola G, Prudenzano AMP, Rainero I, Sances G, Abagnale C, Caponnetto V, De Cesaris F, Frattale I, Guaschino E, Marcinnò A, Ornello R, Pistoia F, Putortì A, Roca ME, Roveta F, Lupi C, Trojano M, Pierelli F, Geppetti P, Sacco S, de Tommaso M (2020). Investigating the effects of COVID-19 quarantine in migraine: an observational cross-sectional study from the Italian National Headache Registry (RICe). Front Neurol.

[13] Del Boca D, Oggero N, Profeta P, Rossi M (2020). Women's and men's work, housework and childcare, before and during COVID-19. Rev Econ Househ.

[14] Time. A Market Crash Was Coming, Coronavirus Was Just the Spark [online]*.* Available at: https://time.com/5793506/a-stock-market-crash-was-coming-coronavirus-was-just-the-spark

[15] American Migraine Foundation. Managing Migraine During COVID-19. Available at: https://americanmigrainefoundation.org/covid-19-resources/

[16] Ali A (2020). Delay in Onabotulinumtoxin a treatment during the COVID-19 pandemic-perspectives from a virus hotspot. Headache..

[17] Di Stefano V, Ornello R, Gagliardo A, Torrente A, Illuminato E, Caponnetto V, Frattale I, Golini R, Di Felice C, Graziano F, Caccamo M, Ventimiglia D, Iacono S, Matarazzo G, Armetta F, Battaglia G, Firenze A, Sacco S, Brighina F (2021). Social distancing in chronic migraine during the COVID-19 outbreak: results from a multicenter observational study. Nutrients..

[18] Lipton RB, Buse DC, Hall CB, Tennen H, Defreitas TA, Borkowski TM, Grosberg BM, Haut SR (2014). Reduction in perceived stress as a migraine trigger: testing the "let-down headache" hypothesis. Neurology.

